# Professional competencies required for Editors of Biomedical Journals

**DOI:** 10.12669/pjms.335.13967

**Published:** 2017

**Authors:** Shaukat Ali Jawaid, Masood Jawaid

**Affiliations:** 1 Shaukat Ali Jawaid Chief Editor, Pakistan Journal of Medical Sciences, Karachi. Pakistan; 2 Masood Jawaid Associate Editor, Pakistan Journal of Medical Sciences, Karachi. Pakistan

There have been tremendous developments in the field of Journalology during the last two decades and now it has emerged as an important discipline with numerous sub-specialties. Most of the editors have so far learnt this art through on the job training. The professional competencies required to be an Editor of a biomedical journal to act as an effective scientific editor has a very broad range and often it is not possible for an individual to fulfill all the requirements and he/she has to depend on the teamwork in which people with expertise in different areas put together their efforts to produce a good quality peer reviewed biomedical journal. They are most often members of the Editorial Board or work in an advisory capacity. The editors are supposed to have some knowledge in areas like research methods, publication ethics, different peer review processes, their advantages and disadvantages, how to protect integrity of their journal/professionalism, they must possess sound knowledge to be able to make good decisions, proficiency in language skills and indexing of the journal in different databases. All this certainly is beyond the capability of an individual and most often these editors have to rely on their other team members.

Associations of Medical Editors in different countries have been organizing different workshops, training courses for their members. WHO EMRO also organized a training course for editors at Shiraz University of Medial Sciences, Shiraz Islamic Republic of Iran from November 14-17^th^ in 2011. Basically it was meant to train the trainers hence one editor was invited from all the EMRO countries. The first author was one of the participants in this course from Pakistan and as per WHO mandatory requirements, on return to Pakistan, organized the training course for the editors under the auspicious of Pakistan Association of Medical Editors (PAME). These courses were organized at Karachi, Lahore, Rawalpindi-Islamabad and Peshawar.

World Association of Medical Editors (WAME) came up with a Syllabus for prospective and newly appointed editors^1^ a few years ago. It gives details about the responsibilities of Editors, What potential Editors should know before accepting the Position. Editorial decision making and communication with authors, editorial process, publication policies, manuscript evaluation, different types of articles besides other responsibilities of the Editors.

For Pakistan we suggested starting a Certificate Course in Medical Journalism^2^ and this suggestion was also presented at the EMAME Conference. Later at the 6th EMAME conference held at Shiraz in 2015, after a formal meeting between Pakistani editors attending the conference, it was proposed to start a Master’s course in Medical Journalism at University of Health Sciences, Lahore. Some detailed discussion took place, which was continued in Pakistan as well. It was agreed that the entire course should be split into five different modules of six months each and the participants should have the option to take up any module after which they will get a certificate. Once someone completes all the five modules and submits a Thesis, he/she would be awarded the Master’s degree. Only preliminary work was undertaken but somehow no substantial progress has been made. The initial enthusiasm seems to have died down. One hopes the project gets revived and we can come up with some practical, feasible, affordable programme. Islamic Republic of Iran has already started a Master’s course in Medical Journalism at Shiraz University of Medical Sciences pioneered by Dr. Behrooz Astaneh Editor of Iranian Journal of Medical Sciences.

Quality and standard of most of the biomedical journals published by professional specialty organizations or medical colleges, universities in Pakistan also leaves much to be desired. One of the important reasons is that most of the faculty members are working as editors in honorary capacity as an additional responsibility. Not only that they do not have any contract appointment and they can be removed anytime. Hence, under such working environment one cannot expect these editors to work with devotion and dedication, concentrating on improving the quality of their journals. It is in the interest of the stake holder’s editors as well as the owner institutions, specialty organizations that the editors should be offered contract appointment clearly defining their role, duties and responsibilities which will be a step in the right direction. The sooner the importance of such a step is realized, better it will be.

More recently Moher et al and their colleagues which included distinguished medical editors from different countries, representatives of various associations of medical editors, scientific publishers have proposed some core competences for scientific editors of biomedical journals.^3^ They took up this project in 2014 and after three years deliberations, came up with a consensus statement and final list of fourteen core competencies for editors of biomedical journals. These are as under:^3^

1. Editors should be able to demonstrate experience and have broad knowledge of the fields covered by their journal.
2. They should be able to synthesize information and views form a wide range of sources and make informed decisions.
3. They should practice lifelong learning related to their role as an editor and within their areas of experience.
4. They should be able to communicate clearly and effectively manage communications and relationship with authors, peer reviewers, other editors, staff, journal owners, readers, publishers and other related individuals, groups.
5. Editors should act with leadership and integrity and be accountable to authors, peer reviewers, fellow editors, readers, journal owners, publishers and other relevant individual and groups.
6. They should demonstrate knowledge related to the integrity of research and publishing and apply best practices in dealing with research or publication misconduct, misbehavior and questionable practices.
7. They should be able to identify and uphold the principles of ethical research involving humans and animals, when appraising manuscripts.
8. They should apply their responsibilities and rights as a journal editor.
9. They should be able to identify and use trustworthy resources.
10. They should be able to select journal contents that reflect the goals and scope of the journal.
11. They should be capable of analyzing journal policies, practices and performance metrics to improve journal performance.
12. Editors should be able to evaluate the scientific rigor and integrity of manuscripts and make editorial decisions after consideration of reviewers and other editor’s comments.
13. They should be capable of applying best practices for research and other manuscript presentation when evaluating and requesting revision of manuscripts.
14. They should be able to manage and assure the integrity of the peer review process.

The above fourteen broad core competences includes different sub-sets like identifying situations where skills required exceeds their level of competency and they need to seek advice from others, exercising sound judgment in making decisions, reconsider decisions when necessary and respond promptly, set personal learning goals, ensure clear instructions for authors and peer reviewers, effective use of all communication channels i.e. correspondence, e mail and social media, mentor, educate train and provide feedback to other editor colleagues, monitor and safeguard fairness and timeliness, know what constitutes a publication ethics, identify conflict of interest on the part of authors and peer reviewers, editors and publishers, manage redundant or duplicate submissions, identify and apply principles of confidentiality, comply with licensing and copy right regulations, ensure best practices as regards advertising policy, identify and use resources for technical editing for authors, editors and peer reviewers, analyze journal performance, formal rational opinion about the submitted manuscripts, practice triage of manuscripts thoroughly and in a timely manner, apply best practices in evaluating citation and references, describe different models of peer review, select peer reviewers who are competent and identify those with conflict of interest, provide feedback to the reviewers, express gratitude to the reviewers besides regularly monitoring and auditing the journal’s performance looking at the acceptance, rejection of manuscripts. They should not hesitate to review their decisions if sound explanation is given with proper references by authors whose manuscripts are first rejected during peer review.

The whole thing is continuously evolving and in the years to come, it is expected that proper training manuals may be developed and a core competency based curriculum will be finalized. Later on the evaluation of these competencies will be possible which could be in the form of some certification. Moher and their colleagues in their consensus statement has also suggested the above in short term and long term goals.^3^ In short we are heading towards exciting times and more experienced, well trained editors will ensure much improved quality of manuscripts accepted for publication and better standard of their journals. No one will be able to accomplish all the above working in a part time capacity and each journal will have to have a minimum of full time staff with clearly defined duties, responsibilities working under a Chief Editor who will be eventually responsible for timely publication, adherence to publication ethics and ensuring integrity of their respective publications.
